# Arsenic Trioxide Inhibits Cell Growth and Induces Apoptosis through Inactivation of Notch Signaling Pathway in Breast Cancer

**DOI:** 10.3390/ijms13089627

**Published:** 2012-08-02

**Authors:** Jun Xia, Youjian Li, Qingling Yang, Chuanzhong Mei, Zhiwen Chen, Bin Bao, Aamir Ahmad, Lucio Miele, Fazlul H Sarkar, Zhiwei Wang

**Affiliations:** 1 Department of Biochemistry and Molecular Biology, Bengbu Medical College, Bengbu 233030, China; E-Mails: xiajunbbmc@126.com (J.X.); meichzh@sina.com (C.M.); chenzhiwen1952@126.com (Z.C.); 2 Laboratory Medicine, Taixing People’s Hospital, Taizhou 225400, China; E-Mail: liyoujian751215@163.com; 3 Research Center of Clinical Laboratory Science, Bengbu Medical College, Bengbu 233030, China; E-Mail: yqlmimi@163.com; 4 Department of Pathology and Oncology, Karmanos Cancer Institute, Wayne State University, Detroit, MI 48201, USA; E-Mails: baob@karmanos.org (B.B.); ahmada@karmanos.org (A.A.); fsarkar@med.wayne.edu (F.H.S.); 5 University of Mississippi Cancer Institute, 2500 N State St, Jackson, MS 39216, USA; E-Mail: lmiele@umc.edu; 6 Department of Pathology, Beth Israel Deaconess Medical Center, Harvard Medical School, 330 Brookline Avenue, Boston, MA 02215, USA

**Keywords:** Notch, arsenic trioxide, NF-κB, breast cancer, apoptosis, cell growth

## Abstract

Arsenic trioxide has been reported to inhibit cell growth and induce apoptotic cell death in many human cancer cells including breast cancer. However, the precise molecular mechanisms underlying the anti-tumor activity of arsenic trioxide are still largely unknown. In the present study, we assessed the effects of arsenic trioxide on cell viability and apoptosis in breast cancer cells. For mechanistic studies, we used multiple cellular and molecular approaches such as MTT assay, apoptosis ELISA assay, gene transfection, RT-PCR, Western blotting, and invasion assays. For the first time, we found a significant reduction in cell viability in arsenic trioxide-treated cells in a dose-dependent manner, which was consistent with induction of apoptosis and also associated with down-regulation of Notch-1 and its target genes. Taken together, our findings provide evidence showing that the down-regulation of Notch-1 by arsenic trioxide could be an effective approach, to cause down-regulation of Bcl-2, and NF-κB, resulting in the inhibition of cell growth and invasion as well as induction of apoptosis. These results suggest that the anti-tumor activity of arsenic trioxide is in part mediated through a novel mechanism involving inactivation of Notch-1 and its target genes. We also suggest that arsenic trioxide could be further developed as a potential therapeutic agent for the treatment of breast cancer.

## 1. Introduction

Breast cancer is the most common malignancy in women, and the second leading cause of cancer-related mortality in women in the United States [1]. According to cancer statistics for 2012 by the American Cancer Society, approximately 226,870 women will be expected to have breast cancer and around 39,510 will die from it in 2012 [1]. Currently, the therapies for breast cancer include surgery, chemotherapy, radiation, hormonal therapy or combined modalities [2]. Although these treatments have improved the five-year survival rate for breast cancer patients, breast cancer still suffers from long term survival, which could be due to late diagnosis, tumor metastasis, chemo- and radio-resistance, and tumor recurrence, resulting in patient death [2]. This worst outcome in a sub-group of patients suggests that it is important to identify newer and novel therapeutic agents for improving the treatment outcome with better long term survival of patients diagnosed with breast cancer.

In recent years, it has been documented that Notch signaling pathway is involved in the development and progression of breast cancer [3–6]. It is known that Notch pathway is a conserved ligand-receptor signaling pathway that plays critical roles in cell proliferation, apoptotic cell death, differentiation, invasion, angiogenesis, tumor metastasis and breast cancer stem cell self-renewal in human breast cancer [3,5]. Notch genes encode transmembrance proteins that can be activated upon ligand binding. To date, four Notch receptors (Notch-1, 2, 3, 4) and five ligands (Dll-1, Dll-3, Dll-4, Jagged-1, and Jagged-2) have been identified [7]. Emerging evidence has shown that activated Notch signaling pathway, and over-expression of Notch target genes are commonly observed in breast cancer [8]. Moreover, high expression of Notch receptors and ligands has been found to correlate with poor prognosis in this deadly disease. Specifically, high-level expression of Jagged-1, Notch-1 and Notch-2 has been found to be associated with poor overall survival in human breast cancer [9,10]. Moreover, Jagged-1 expression was found to correlate with recurrence of lymph node-negative breast cancer [11]. Recently, it has been reported that Notch-1 and Notch-4 could serve as prognostic markers in breast cancer [12,13]. Furthermore, multiple studies have demonstrated that Notch signaling pathway plays an important role in chemo-resistance of breast cancer [14]. Therefore, targeting Notch signaling pathway could be a promising strategy to achieve better treatment outcome for breast cancer.

Recent studies have shown that arsenic trioxide (As_2_O_3_), a clinically effective reagent for APL (acute promyelocytic leukemia), inhibited cell growth and induced apoptosis in a variety of human cancers including breast cancer [15–19]. For example, As_2_O_3_ was shown to dramatically reduce the survival of MCF-7 and T47D breast cancer cells via inhibition of estrogen receptor [20]. Another study showed that As_2_O_3_ exhibited inhibitory effects on the proliferation of MCF-7 cells through up-regulation of p53 tumor suppressor protein and down-regulation of Bcl-2 protein level [17]. Recently, it was found that As_2_O_3_ suppressed MCF-7 cell growth through induction of p21 and p27 tumor suppressor proteins [21]. However, the comprehensive molecular mechanism(s) by which As_2_O_3_ inhibits cell growth and induces apoptosis remains largely elusive. Thus, exploring the molecular physiological properties of As_2_O_3_ could lead to its novel therapeutic use for the treatment of breast cancer.

## 2. Results

### 2.1. As_2_O_3_ Inhibited Breast Cancer Cell Growth

**Figure 1:**
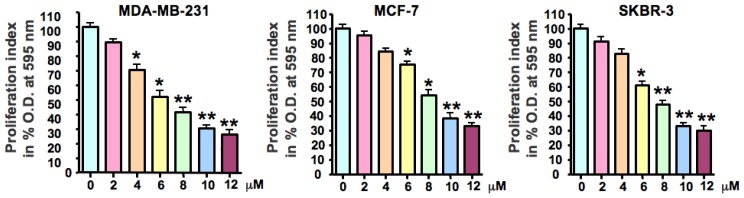
Effect of As_2_O_3_ on breast cancer cell growth. Cells were seeded in 96-well plates at 5000 cells per well and treated with varied concentrations of As_2_O_3_ for 72 h. After treatment, MTT solution was added and incubated further for 2 h. MTT formazan formed by metabolically viable cells was dissolved in isopropanol, and absorbance was measured at 595 nm on a plate reader (TECAN). Each value represents the mean ± SD (*n* = 6) of three independent experiments. * *p* < 0.05, ** *p* < 0.01, compared to the control.

First, we tested the growth inhibitory effects of As_2_O_3_ using the MTT assay in three human breast cancer cell lines, MDA-MB-231, MCF-7, and SKBR-3. As expected, treatment of breast cancer cells for 72 h with 2, 4, 6, 8, 10, and 12 μM of As_2_O_3_ led to cell growth inhibition in a dose-dependent manner in all three breast cancer cell lines (Figure 1). The IC_50_ that caused 50% inhibition of cell growth for three breast cancer cell lines was found around 8 μM.

### 2.2. As_2_O_3_ Induced Apoptosis in Breast Cancer Cell Lines

**Figure 2:**
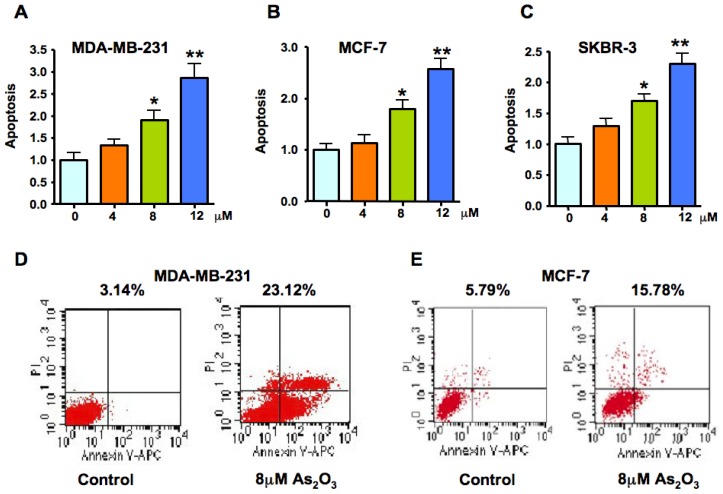
Effect of As_2_O_3_ on breast cancer cell apoptosis. Cell death assay for measuring apoptosis induced by As_2_O_3_ was done in MDA MB-231 (**A**), MCF-7 (**B**) and SKBR-3 (**C**) cells treated with different doses of As_2_O_3_ for 72 h. Apoptosis was measured by Histone-DNA ELISA method. Values are reported as mean ± SD. * *p* < 0.05, ** *p* < 0.01, compared to the control. (**D**, **E**) MDA MB-231 and MCF-7 cells were treated with 8 μM As_2_O_3_ for 72 h. Annexin V/PI staining was performed to detect the apoptosis.

MDA-MB-231, MCF-7, and SKBR-3 cells were treated with 4, 8 and 12 μM As_2_O_3_ for 72 h. After treatment, the degree of apoptosis was measured in all three breast cancer cell lines. We found that the As_2_O_3_ treatment induced apoptosis in dose-dependent manner in all three breast cancer cells (Figure 2A–C). To further confirm the results from our histone/DNA ELISA data, we used Annexin V/PI staining. As demonstrated in Figure 2D,E, 8 μM As_2_O_3_ at 72 h induced apoptosis in breast cancer cell lines. These results clearly suggested that As_2_O_3_ treatment caused a statistically significant increase in the percentage of apoptotic cells in breast cancer cell lines.

### 2.3. As_2_O_3_ Suppressed Breast Cancer Cell Invasion

**Figure 3:**
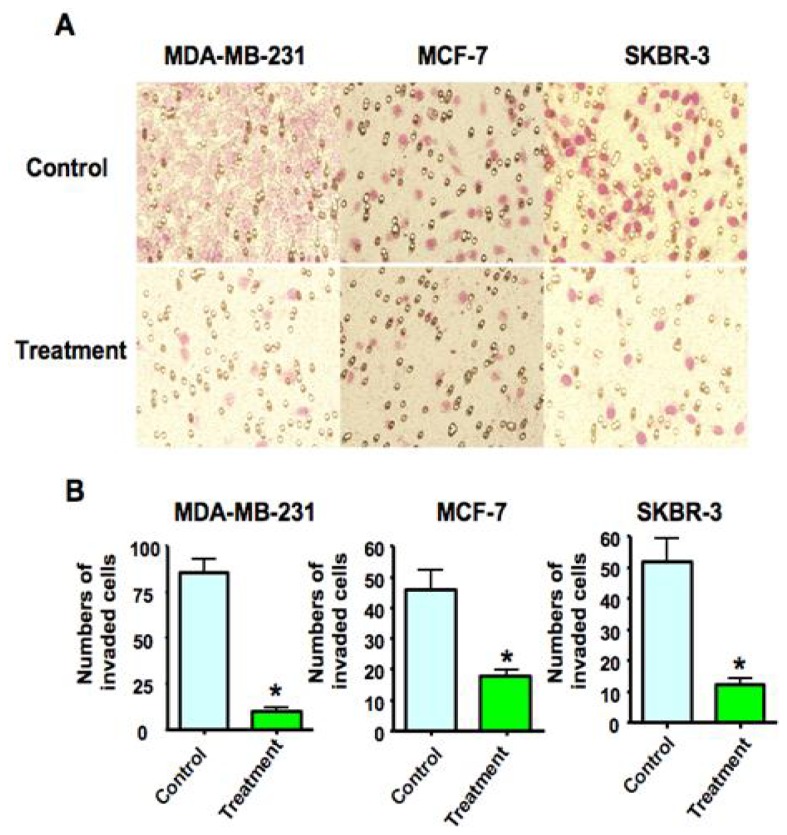
Effect of As_2_O_3_ on breast cancer cell invasion. (**A**) Invasion assay showing that As_2_O_3_-treated cells resulted in low penetration through the Matrigel-coated membrane, compared with control cells. (**B**) Numbers of the invaded cells and these numbers indicate the ability of cell invasion. * *p* < 0.05 compared to the control.

Consistent with the anti-invasive role of As_2_O_3_, we found that 8 μM As_2_O_3_ resulted in decreased penetration of breast cancer cells through the matrigel-coated membrane compared with the control cells. Further quantitation of the numbers of invaded breast cancer cells was significantly decreased after As_2_O_3_ treatment compared to control cells (Figure 3). It is important to note that 8 μM As_2_O_3_ did not inhibit the cell growth at 24 h (data not shown), suggesting that the decrease in cell invasion is not due to a drop in cell numbers.

### 2.4. As_2_O_3_ Inhibited the Notch-1 Expression in Breast Cancer Cells

**Figure 4:**
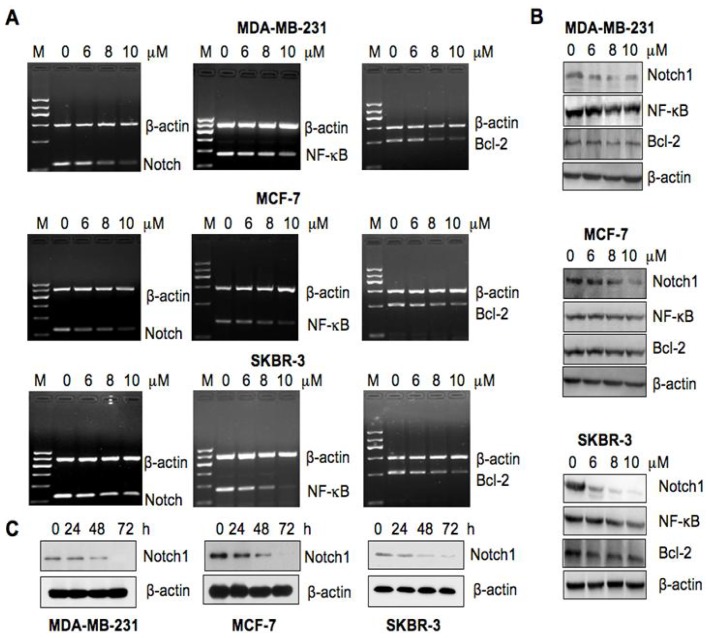
Inhibition of Notch-1 signaling pathway by As_2_O_3_ in breast cancer cells. (**A**) The Notch-1, Bcl-2 and NF-κB mRNA were detected by RT-PCR in breast cancer cells treated with varied concentrations of As_2_O_3_ for 72 h; (**B**) The Notch-1, Bcl-2, and NF-κB proteins were measured by Western blotting analysis in breast cancer cells treated with varied concentrations of As_2_O_3_ for 72 h; (C) The Notch-1 expression was detected by Western blotting analysis in breast cancer cells treated with 8 μM As_2_O_3_ for different times.

Next, we investigated whether As_2_O_3_ exerts its anti-tumor activity through down-regulation of Notch signaling pathway. The expression of Notch-1 in As_2_O_3_-treated breast cancer cells was assessed by RT-PCR and Western blotting analysis, respectively. We found that both Notch-1 mRNA and protein levels were down-regulated after As_2_O_3_ treatment in all three breast cancer cell lines (Figure 4A,B). More importantly, we observed that As_2_O_3_ inhibited the Notch-1 expression at 48 h (Figure 4C), suggesting that Notch-1 decrease is probably causative for As_2_O_3_-induced apoptosis.

### 2.5. As_2_O_3_ Inhibited the Expression of Notch-1 Downstream Genes

Next, we investigated whether As_2_O_3_ treatment could cause down-regulation of *Notch-1* downstream genes. It has been well characterized that NF-κB and Bcl-2 are two key downstream targets of Notch-1 [22,23]. Therefore, we assessed the expression of NF-κB and Bcl-2 at both mRNA and protein levels. Our results showed that As_2_O_3_ suppressed the expression of NF-κB and Bcl-2 both at the mRNA and protein levels in three breast cancer cells (Figure 4).

### 2.6. Down-Regulation of Notch-1 Expression by SiRNA and the Effect of As_2_O_3_ Treatment

**Figure 5:**
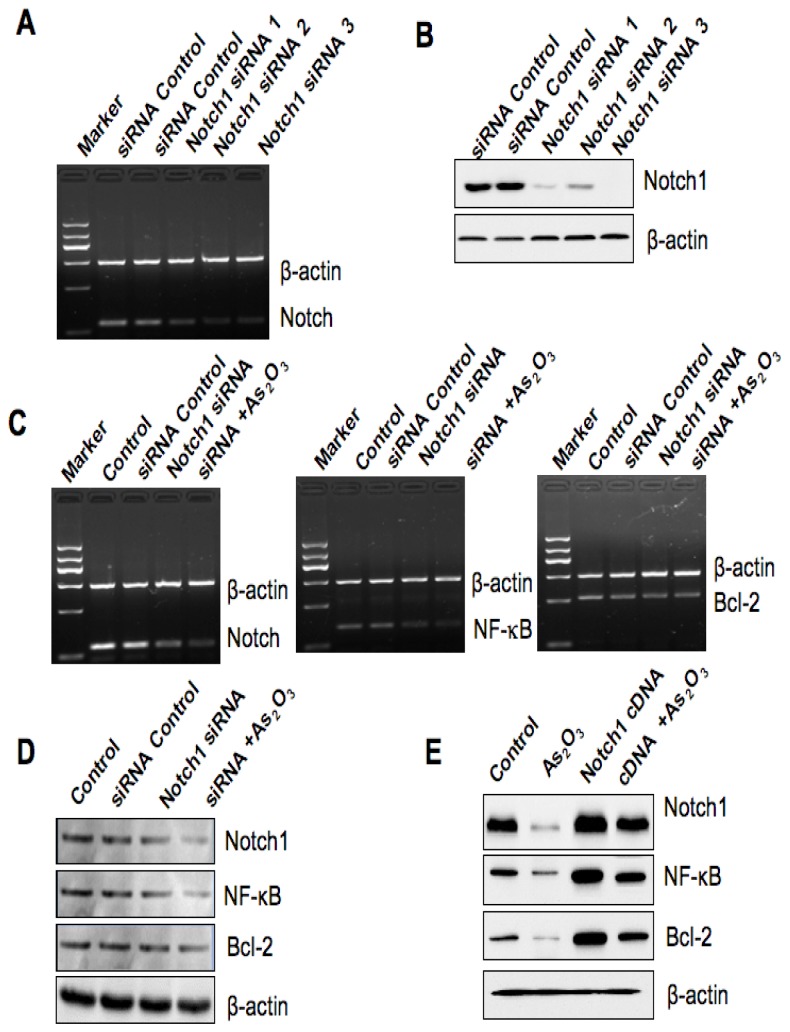
The efficacy of transfection by Notch-1 siRNA and Notch-1 cDNA in SKBR-3 cells. (**A**–**D**) The expression of Notch-1 was detected by RT-PCR and Western blotting, respectively, to check the Notch-1 siRNA transfection efficacy. (**E**) The expression of Notch-1 was detected by Western blotting for assessing the Notch-1 cDNA plasmid transfection efficacy.

**Figure 6:**
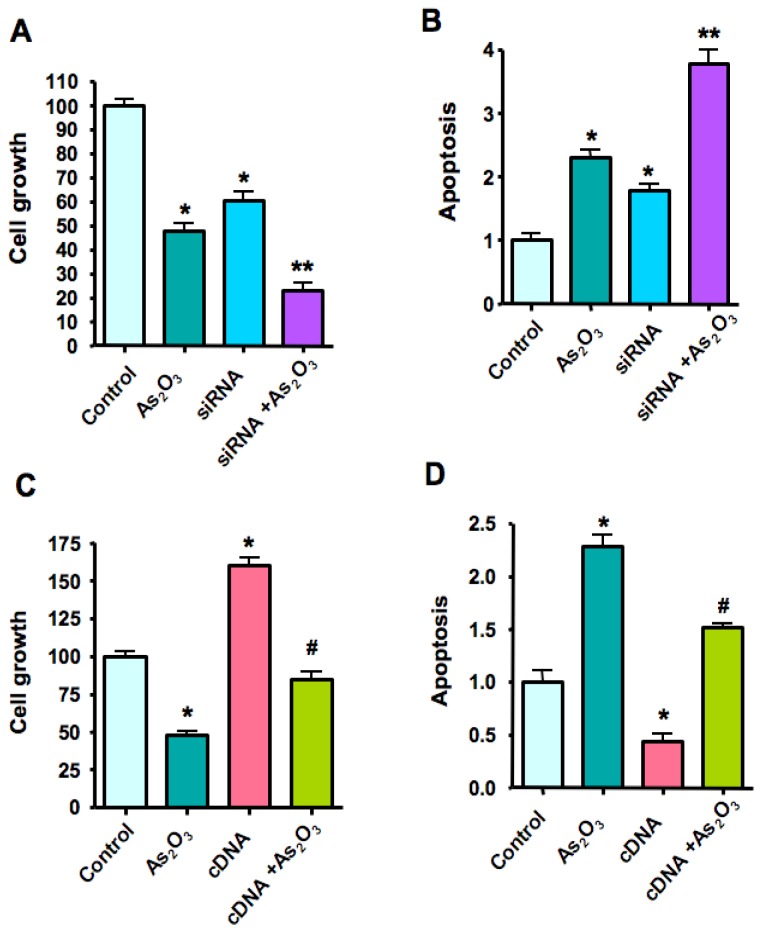
Notch-1 siRNA promoted, but Notch-1 cDNA reduced, As_2_O_3_-induced cell growth inhibition and apoptosis in SKBR-3 breast cancer cells (**A**–**B**). Left panel, down-regulation of Notch-1 by siRNA significantly inhibited SKBR-3 breast cancer cell growth. 8 μM As_2_O_3_ plus Notch-1 siRNA inhibited cell growth to a greater degree compared to As_2_O_3_ alone. Right panel, down-regulation of Notch-1 expression significantly increased apoptosis induced by As_2_O_3_. Notch-1 siRNA transfected cells were significantly more sensitive to spontaneous and As_2_O_3_-induced apoptosis (**C**–**D**). Over-expression of Notch-1 by cDNA transfection rescued As_2_O_3_-induced cell growth and abrogated As_2_O_3_-induced apoptosis to a certain degree. * *p* < 0.05, compared with the control; ** *p* < 0.05, compared with As_2_O_3_ treatment alone and Notch-1 siRNA transfection alone. # *p* <0.05, compared with As_2_O_3_ treatment alone and Notch-1 cDNA transfection alone.

To study the functional relevance of As_2_O_3_-mediated alteration of Notch-1 expression in breast cancer cells, we used Notch-1 siRNA to deplete the endogenous expression of Notch-1 and subsequently examined the effect of Notch-1 siRNA on cell growth and apoptosis followed by 8 μM As_2_O_3_ treatment in SKBR-3 cells. The reason we selected SKBR-3 cell line for further study is that these cells have a higher expression, but not the highest, of Notch-1 in multiple breast cancer cell lines [24]. The efficacy of Notch-1 siRNA for depletion of Notch-1 mRNA and protein was validated by RT-PCR and Western blotting analysis, respectively (Figure 5). Moreover, consistent with this, we found that the expression of Notch-1 target gene NF-κB and Bcl-2 was also decreased after depletion of Notch-1 (Figure 5). Our results also showed that depletion of Notch-1 by siRNA transfection caused cell growth inhibition and apoptosis (Figure 6). More importantly, As_2_O_3_ treatment plus Notch-1 siRNA retarded cell growth to a greater degree compared to As_2_O_3_ alone. Furthermore, breast cancer cells with Notch-1 siRNA treatment were more sensitive to As_2_O_3_-induced apoptosis (Figure 6).

### 2.7. Over-Expression of *Notch-1* by cDNA Transfection Reduced As_2_O_3_-Induced Cell Growth Inhibition and Apoptosis

Breast cancer cells were transfected with Notch-1 cDNA or empty vector control (pcDNA3). The expression of Notch-1 and its target genes was measured to confirm that Notch-1 cDNA transfection led to up-regulation of Notch-1 pathway (Figure 5). Moreover, over-expression of Notch-1 promoted cell growth and protected from apoptosis (Figure 6). Furthermore, over-expression of *Notch-1* by cDNA transfection rescued As_2_O_3_-induced cell growth inhibition and reduced As_2_O_3_-induced apoptosis to 60%–70%.

## 3. Discussion

In the current study, we investigated the effects of As_2_O_3_ on cell proliferation and apoptosis in breast cancer cells. We found that As_2_O_3_ caused cell growth inhibition and induced apoptosis. Moreover, we found a significant down-regulation of Notch-1 expression and the expression of its downstream genes after As_2_O_3_ treatment. Furthermore, our results demonstrated that As_2_O_3_-induced down-regulation of Notch-1 is associated with As_2_O_3_-mediated cell growth inhibition and apoptosis. These results suggest that down-regulation of Notch-1 could be a novel strategy for the treatment of breast cancer by As_2_O_3_.

Recent studies have demonstrated that Notch signaling pathway is involved in the development and progression of breast cancer [3–6]. Several studies also suggested that Notch-1 signaling pathway is involved in drug resistance in a variety of human cancers including breast cancer [14]. For example, down-regulation of Notch-1 signaling pathway increased chemosensitivity to several chemotherapeutic drugs such as taxotere, doxorubicin, and tamoxifen, indicating that Notch signaling pathway could be a novel target for overcoming drug-resistance in breast cancer [25–28]. Moreover, it has been reported that the fate of breast cancer stem cells is controlled by Notch pathway in breast cancer [29–33]. Taken together, inactivation of Notch pathway could be a promising strategy for achieving better treatment for breast cancer.

Since Notch signaling is activated via the activity of γ-secretase, development of γ-secretase inhibitors (GSIs) could be used for cancer therapy. Several GSIs have been reported to inhibit cell growth, increase apoptosis, and reduce cell invasion in breast cancer [25,28,34]. Moreover, it has been found that GSIs reduced the formation of brain metastasis from breast cancer through reduction of breast CSCs (Cancer stem cells) [32]. In a recent study, Rizzo *et al.* found that inactivating Notch-1 by GSIs could potentiate the effects of tamoxifen in breast cancer cell growth *in vitro* and *in vivo* [28]. In addition, GSIs re-sensitized trastuzumab-resistant BT474 cells to trastuzumab, suggesting that Notch-1 might play a novel role in resistance to trastuzumab [25]. More importantly, Kondratyev *et al.* showed that GSIs could eliminate CSCs and inhibited the self-renewal and proliferation of breast CSCs [31]. Although GSIs have the advantage of relative ease of administration, oral bioavailability and low cost, GSIs have unwanted toxicity such as cytotoxicity in the gastrointestinal tract [35]. In addition, GSIs are relatively nonselective drugs because they block the cleavage of all four Notch receptors and other multiple γ-secretase substrates [35]. Therefore, it is obvious that discovery of new compounds to target Notch signaling pathway is needed.

In recent years, As_2_O_3_, a compound used in traditional Chinese medicine for many years, has been reported to improve standard care for APL [36]. Consistent with the anti-tumor activity of As_2_O_3_ in APL, studies from many independent groups also showed that As_2_O_3_ inhibited cancer cell growth and induced apoptosis in a variety of human cancers [37–40]. In this study, we used three human breast cancer cell lines, MDA-MB-231, MCF-7, and SKBR-3, which expressed high levels of Notch-1, and we found that As_2_O_3_ elicited a significant effect on growth inhibition and induction of apoptotic cell death in breast cancer cells. Although As_2_O_3_ has been found to inhibit cancer cell invasion in multiple human cancer cell lines [27,41–43], anti-invasive function of As_2_O_3_ in breast cancer cells has not been reported. Therefore, we determined the effects of As_2_O_3_ on breast cancer cell invasion. As we expected, As_2_O_3_ inhibited the breast cancer cell invasion. In order to further determine the molecular mechanism by which As_2_O_3_ induced cell growth inhibition as well as apoptosis and inhibited invasion in breast cancer cell lines, alterations in the cell survival pathway were explored. It has been well documented that Notch signaling is up-regulated in many human cancers including breast cancer and plays a critical role in cell growth and invasion, and suppression of apoptosis [3,5]. Consistent with one study that As_2_O_3_ inhibited Notch-1 and its target gene Hes-1 in gliomas [44], we found that As_2_O_3_ down-regulated the expression of Notch-1 and its target genes in breast cancer cell lines. Importantly, depletion of Notch-1 by siRNA together with As_2_O_3_ treatment caused cell growth inhibition and apoptosis to a greater degree in breast cancer. Interestingly, over-expression of Notch-1 by cDNA transfection reduced As_2_O_3_-induced cell growth inhibition and apoptosis. Based on these findings, we believe that inactivation of Notch-1 signaling by As_2_O_3_ leads to inactivation of its target gene expression, which could be mechanistically linked with As_2_O_3_-mediated tumor suppressor function.

## 4. Experiment Section

### 4.1. Cell Lines and Experimental Reagents

Human breast cancer cell lines, MDA-MB-231, MCF-7 and SKBR-3 were obtained from American Type Culture Collection (Manassas, VA) and used in this study. Primary antibodies for Notch-1, Bcl-2, and NF-κB p65 were purchased from Santa Cruz Biotechnology (Santa Cruz, CA). The monoclonal antibody to β-actin was bought from Sigma-Aldrich (St. Louis, MO). All secondary antibodies were obtained from Pierce (Rockford, IL). Lipofectamine 2000 was purchased from Invitrogen (Carlsbad, CA). Protease inhibitor cocktail, As_2_O_3_, 3-(4,5-dimethylthiazol-2-yl)-2,5-diphenyltetrazolium bromide (MTT), and all other chemicals were obtained from Sigma-Aldrich. As_2_O_3_ was dissolved in 1 mM NaOH to make a 10 mM stock solution and was added directly to the media at different concentrations.

### 4.2. Cell Growth Inhibition Studies by MTT Assay

Breast cancer cells (5000) were seeded in 96-well culture plates and treated with different concentrations of As_2_O_3_ (2, 4, 6, 8, 10, 12 μM) diluted from the stock solution. After 72 h, MTT solution was added and incubated for 2 h. MTT assay for determining cell growth inhibition by As_2_O_3_ was performed as described earlier [45].

### 4.3. Histone-DNA Enzyme-Linked Immunosorbent Assay (ELISA) for Detecting Apoptosis

Since the loss of cell viability could be due to the induction of apoptosis, we further examined the effects of As_2_O_3_ treatment on apoptotic cell death using Histone-DNA ELISA method. The cell apoptosis ELISA detection method (Roche, Palo Alto, CA) was used according to the manufacturer’s protocol. Briefly, after different concentration of As_2_O_3_ (4, 8, 12 μM) treatment for 72 h, the cytoplasmic Histone/DNA fragments from treated cells were extracted and bound to anti-Histone antibody for detection of apoptosis as described earlier [46].

### 4.4. Annexin V-FITC Method for Apoptosis Analysis

Annexin V-FITC apoptosis detection kit (BD, San Jose, USA) was used to measure the apoptotic cells. Briefly, cells were treated with 8 μM As_2_O_3_ for 72 h and then trypsinized, washed twice with ice-cold PBS and the number of apoptotic cells was analyzed as described before [47].

### 4.5. Cell Invasion Assay

The invasive activity of the MDA-MB-231, MCF-7 and SKBR-3 cells followed by 8 μM As_2_O_3_ treatment was detected using the BD BioCoat Tumor Invasion Assay System (BD Biosciences, Bedford, MA). Briefly, breast cancer cells with serum-free medium supplemented with 8 μM As_2_O_3_ were seeded into the upper chamber of the system. Bottom wells in the system were filled with complete medium and 8 μM As_2_O_3_. After 24 h of incubation, the cells in the upper chamber were removed, and the cells that had invaded through the matrigel matrix membrane were stained with Wright-Giemsa for 15 min. These stained invasive cells were photographed and counted under a microscope.

### 4.6. Reverse Transcription-PCR (RT-PCR) Analysis for Gene Expression Studies

The total RNA from As_2_O_3_-treated cells was isolated by Trizol (Invitrogen, Carlsbad, CA) and purified by RNeasy Mini Kit and RNase-free DNase Set (QIAGEN, Valencia, CA) according to the manufacturer’s protocols. One microgram of total RNA from each sample was subjected to first strand cDNA synthesis using TaqMan reverse transcription reagents kit (Applied Biosystems, Foster City, CA). RT reaction was performed at 25 °C for 10 min, followed by 48 °C for 30 min and 95 °C for 5 min. PCR reaction was performed at 94 °C for 30 s, followed by 48 °C for 30 s and 72 °C for 40 s. The primers used in the PCR reaction were as follows: NOTCH-1: 5′-CGA CGT CAA CGC CGT AGA T-3′ and 5′-CTC CTC CCT GTT GTT CTG CAT AT-3′; NF-κB: 5′-AGG ACA TAT GAG ACC TTC AAG AGC-3′ and 5′-CTC ATC ATA GTT GAT GGT GCT CAG-3′; Bcl-2: 5′-GGC GCA CGC TGG GAG AAC-3′ and 5′-TAG CGG CGG GAG AAG TCG TC-3′. GAPDH: 5′-CAA GGT CAT CCA TGA CAA CTT TG-3′ and 5′-GTC CAC CAC CCT GTT GCT GTA G-3′.

### 4.7. Western Blot Analysis

Cells were lysed in EBC (50 mM Tris pH 7.5, 120 mM NaCl, 0.5% NP-40) buffer supplemented with protease inhibitors (Complete Mini, Roche) and phosphatase inhibitors (phosphatase inhibitor cocktail set I and II, Calbiochem). The protein concentrations of the lysates were determined using the Bio-Rad assay system (Bio-Rad, Hercules, CA). Total proteins were fractionated using SDS-PAGE and immuno-blotted with indicated antibodies as described earlier [47].

### 4.8. Plasmids and Transfections

Notch-1 siRNA and siRNA control were obtained from Santa Cruz Biotechnology (Santa Cruz, CA). The Notch-1 cDNA plasmid encoding the Notch-1 intracellular domain was described previously [48]. Human breast cancer cells were transfected with Notch-1 siRNA and cDNA, respectively, using Lipofectamine 2000 as described earlier [49].

### 4.9. Densitometric and Statistical Analysis

The statistical significance of differential findings between experimental groups and control groups was statistically evaluated using GraphPad StatMate software (GraphPad Software, Inc., San Diego, CA). *p* values lower than 0.05 were considered statistically significant.

## 5. Conclusions

In conclusion, our current findings suggest that As_2_O_3_ may function as a Notch-1 inhibitor, resulting in cell growth inhibition and induction of apoptosis. The inactivation of Notch-1 by As_2_O_3_ decreased the expression of Bcl-2 and NF-κB, which likely leads to the inhibition of invasion. However, further investigations are required to determine the exact molecular mechanism(s) underlying As_2_O_3_-mediated tumor suppression together with conventional therapeutics. Furthermore, *in vivo* animal studies together with clinical trials are necessary to confirm the tremendous potential of As_2_O_3_ for the treatment of human breast cancer.
