# Xia, J.; *et al.*, Arsenic Trioxide Inhibits Cell Growth and Induces Apoptosis through Inactivation of Notch Signaling Pathway in Breast Cancer. *Int. J. Mol. Sci.* 2012, *13*, 9627–9641

**DOI:** 10.3390/ijms150814907

**Published:** 2014-08-22

**Authors:** Jun Xia, Youjian Li, Qingling Yang, Chuanzhong Mei, Zhiwen Chen, Bin Bao, Aamir Ahmad, Lucio Miele, Fazlul H. Sarkar, Zhiwei Wang

**Affiliations:** 1 Department of Biochemistry and Molecular Biology, Bengbu Medical College, Bengbu 233030, China; E-Mails: xiajunbbmc@126.com (J.X.); meichzh@sina.com (C.M.); chenzhiwen1952@126.com (Z.C.); 2 Laboratory Medicine, Taixing People’s Hospital, Taizhou 225400, China; E-Mail: liyoujian751215@163.com; 3 Research Center of Clinical Laboratory Science, Bengbu Medical College, Bengbu 233030, China; E-Mail: yqlmimi@163.com; 4 Department of Pathology and Oncology, Karmanos Cancer Institute, Wayne State University, Detroit, MI 48201, USA; E-Mails: baob@karmanos.org (B.B.); ahmada@karmanos.org (A.A.); fsarkar@med.wayne.edu (F.H.S.); 5 University of Mississippi Cancer Institute, 2500 N State St., Jackson, MS 39216, USA; E-Mail: lmiele@umc.edu; 6 Department of Pathology, Beth Israel Deaconess Medical Center, Harvard Medical School, 330 Brookline Avenue, Boston, MA 02215, USA

The authors wish to change Figure 5D of the paper published in IJMS [1]. In Figure 5D, the bands for NF-κB and Bcl-2 are similar with Notch-1 bands. The authors have carefully checked the original files and found that it is an inadvertent mistake in the published version of Figure 5D. Figure 5 is revised as follows. The authors would like to apologize for any inconvenience caused to the readers by these changes.

**Figure 5:**
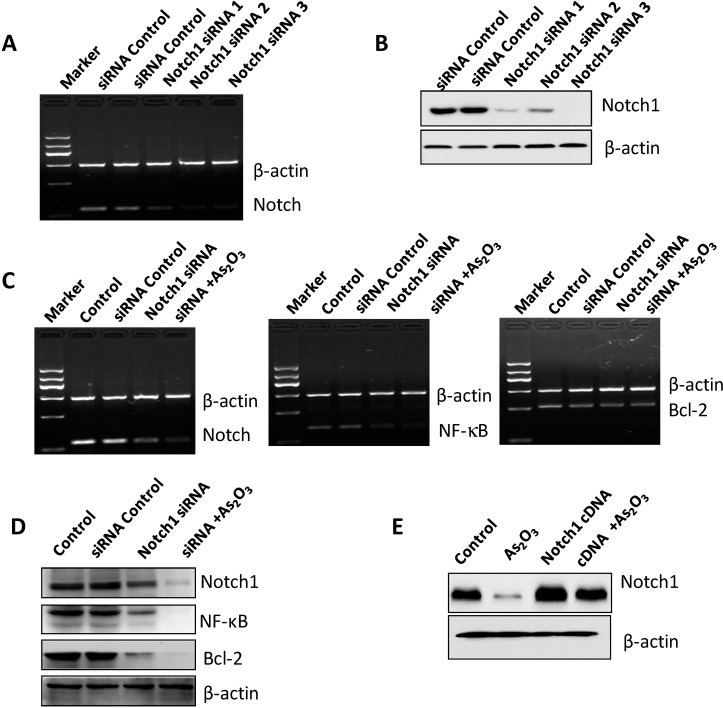
The efficacy of transfection by Notch-1 siRNA and Notch-1 cDNA in SKBR-3 cells. **A**-**D**: The expression of Notch-1 was detected by RT-PCR and Western blotting, respectively, to check the Notch-1 siRNA transfection efficacy; **E**: The expression of Notch-1 was detected by Western blotting for assessing the Notch-1 cDNA plasmid transfection efficacy.
